# Extended lag phases and variable antibiotic tolerance in β-lactam-induced small colony variants of *Enterococcus faecalis*

**DOI:** 10.1186/s12866-026-05403-y

**Published:** 2026-07-17

**Authors:** Lara Thieme, Kamran A. Mirza, Mehri Azimi, Nicole Enslinger, Mara Lohde, Mateusz Jundzill, Christian Brandt, Mathias W. Pletz, Oliwia Makarewicz

**Affiliations:** 1 Institute for Infectious Diseases and Infection Control, Jena University Hospital, Friedrich-Schiller-University Jena, Am Klinikum 1, Jena, 07747 Germany; 2 Leibniz Center for Photonics in Infection Research, Jena, 07743 Germany; 3 Department Cellular and Molecular Medicine, Department of Medicine, University of Ottawa, Ottawa, ON K1N 9A9 Canada; 4 InfectoGnostics Research Campus, Jena, 07743 Germany; 5 Dynamics42 GmbH, Jena, 07745 Germany

**Keywords:** Endocarditis, Antimicrobial resistance, Chronic infections, Cephalosporins, Synergy, Wax moth

## Abstract

Small colony variants (SCVs) of *Enterococcus faecalis* are associated with persistent infections and have been linked to altered antibiotic susceptibility and tolerance. Here, we investigated SCVs emerging under β-lactam exposure in comparison to corresponding normal colony phenotypes (NCPs). SCVs were generated in vitro under benzylpenicillin, ceftriaxone, and combination therapy and characterized by growth kinetics, antibiotic susceptibility (MIC), killing dynamics (MDK), and bactericidal activity (MBC). Virulence and infection dynamics were further assessed using the *Galleria mellonella* model. SCVs displayed reduced growth rates and prolonged lag phases, which were partially reversible upon subcultivation. β-Lactam exposure induced substantial MIC increases across multiple lineages, including both SCV and NCP variants, indicating that reduced susceptibility represents a general adaptive response rather than a phenotype-specific trait. In contrast, killing dynamics revealed heterogeneous tolerance profiles that were not consistently associated with SCV formation. While selected SCVs exhibited prolonged MDK values consistent with increased tolerance, other variants showed rapid killing despite elevated MICs. MBC values further supported this dissociation, particularly for ceftriaxone, where bactericidal activity remained limited across variants. In the *G. mellonella* model, SCVs showed delayed infection onset but did not uniformly result in reduced virulence. Together, these findings indicate that SCVs in *E. faecalis* represent a heterogeneous and reversible adaptive state. Antibiotic tolerance depends on growth dynamics rather than phenotype alone, while MIC shifts occur independently of SCV formation, highlighting the importance of distinguishing between resistance, tolerance, and phenotypic adaptation.

**Graphical abstract:**

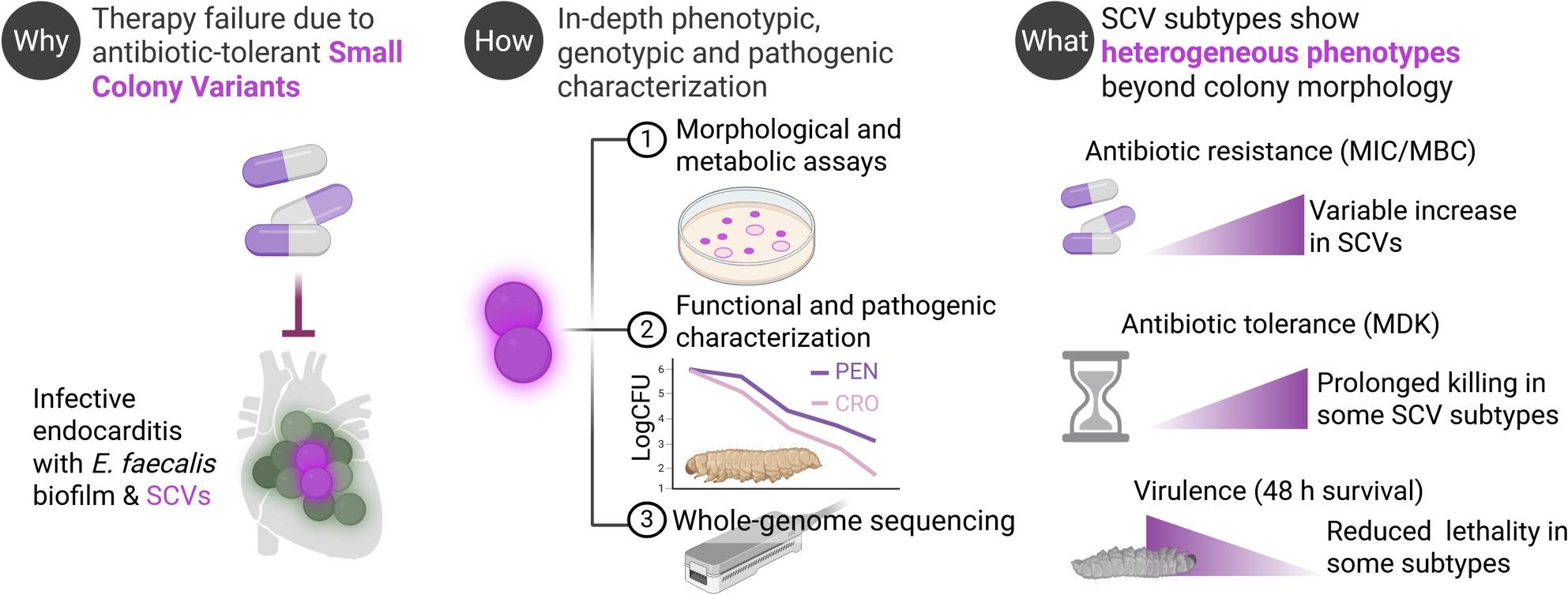

**Supplementary Information:**

The online version contains supplementary material available at 10.1186/s12866-026-05403-y.

## Introduction

The persistence of bacteria in chronic infections can be attributed to multiple factors, including biofilm formation, evasion of host immune responses, and the emergence of resistant and tolerant subpopulations. Together, these mechanisms create major therapeutic challenges in eradicating pathogens [1]. Among these subpopulations, small colony variants (SCVs) are particularly problematic. SCVs are slow-growing bacteria that exhibit distinct alterations in morphology, growth dynamics, and metabolic activity compared to their parental strains [2]. Conventionally, SCVs are identified as pinpoint colonies on agar plates that are significantly smaller than the normal colony phenotype (NCP) [2]. SCVs frequently emerge under environmental selection pressures, including prolonged antibiotic exposure, and contribute to chronic infections by promoting intracellular persistence and metabolic adaptation as particularly shown for *Staphylococcus aureus* [3]. The best-characterized *S. aureus* SCV phenotypes are linked to mutations in the menadione or hemin biosynthesis pathways, both of which are required for cytochrome synthesis [4]. These mutations impair ATP generation and slow bacterial growth, distinguishing SCVs from their parental strains.

SCVs generally do not harbor conventional antibiotic resistance genes but display tolerance to antimicrobials. Resistance and tolerance are distinct survival strategies. Resistant bacteria can proliferate even at high antibiotic concentrations, often due to genetic mechanisms such as efflux pump overexpression, enzymatic drug inactivation (e.g., β-lactamases), or altered drug targets. In contrast, tolerant bacteria survive otherwise lethal concentrations by temporarily slowing essential processes, sacrificing growth until selective pressure is removed, after which growth resumes and infection relapses may occur [5]. Tolerance is usually mediated by nonspecific genetic factors, such as mutations in the electron transport chain, frequently observed in *S. aureus* SCVs [6]. Antibiotic tolerance can phenotypically mimic resistance, as tolerant bacteria may survive prolonged antibiotic exposure without a corresponding increase in minimum inhibitory concentration (MIC). Understanding the mechanisms underlying SCV tolerance is therefore crucial for improving treatment strategies against chronic infections.

Compared to *S. aureus*, much less is known about SCVs in enterococci. Both *Enterococcus faecalis* and *Enterococcus faecium* are important nosocomial pathogens. While *E. faecium* is difficult to treat due to its broad resistance profile, including vancomycin resistance, *E. faecalis* is more frequently isolated clinically, largely due to its strong ability to form biofilms that tolerate antibiotics [7]. *E. faecalis* is increasingly linked to chronic infections, including infective endocarditis, infections of cardiac devices or prosthetic joints, bone and joint infections, and wound infections, particularly in immunocompromised patients [7]. Notably, E. faecalis SCVs have been reported in infective endocarditis and other clinically persistent infection settings, including cases with recurrent or prolonged disease courses [8–10]. Despite these findings, detailed knowledge of enterococcal SCVs remains scarce. To date, only one study published in 2009 has comprehensively described an *E. faecalis* SCV, reporting reduced sugar fermentation, altered morphology, growth behavior, and decreased aminoglycoside susceptibility compared to the parental strain [10].

We previously reported in vitro that E. faecalis biofilms exposed to β-lactam antibiotics, particularly in combination therapy, drive the concentration-dependent emergence of SCVs [11, 12]. The frequency of SCV formation varied by strain and by antibiotic group, with differences between benzylpenicillins and cephalosporins. Clinically, cephalosporins are combined with aminopenicillins for the treatment of *E. faecalis* infective endocarditis, as this regimen provides synergistic activity through complementary targeting of penicillin-binding proteins, despite the intrinsic resistance of enterococci to cephalosporins [13]. Intravenous benzylpenicillin has also been used as an alternative to intravenous ampicillin or amoxicillin in outpatient parenteral antimicrobial therapy, particularly in New Zealand, Australia, and the United States, due to its greater stability in elastomeric continuous infusors [14, 15].

The present study aimed to extend the characterization of antibiotic-induced *E. faecalis* SCVs on phenotypic, genotypic, and pathogenic levels. We compared metabolic and morphological traits of SCVs to previously reported data, evaluated virulence in the *Galleria mellonella* infection model, and performed whole-genome sequencing to identify relevant genetic changes. Our findings provide evidence that antibiotic exposure selects for a heterogeneous set of SCV phenotypes in *E. faecalis*, including a previously unrecognized long-lag subtype.

## Methods

### Selection of E. faecalis strain and antibiotics

The clinical endocarditis isolate *E. faecalis* 26,786 was selected from the cohort of our previous dual β-lactam antibiotic synergy study comparing synergistic effects between ampicillin or benzylpenicillin, respectively, with ceftriaxone [11]. The isolate had shown a strong tendency to form SCVs under antibiotic pressure. Its MIC for benzylpenicillin (MIC_PEN_) was 1 mg/L and for ceftriaxone (MIC_CRO_) 4 mg/L. The anonymous use of the isolate for research purpose was approved by the Ethic Committee of the Jena University Hospital (Germany) under the registration number 2025-3915-BO-B.

The ceftriaxone (TCI Europe, Zwijndrecht, Belgium) and benzylpenicillin (InfectoPharm, Heppenheim, Germany) were prepared immediately before use as stock solutions in sterile deionized water.

### Isolation of small colony variants

Bacterial liquid cultures prepared in either Todd Hewitt (TH) or Mueller-Hinton (MH) broth (both Karl Roth, Karlsruhe, Germany) were challenged overnight with selected single and combined concentrations of benzylpenicillin and ceftriaxone, according to the checkerboard antibiotic synergy set-up described previously [12]. Chosen antibiotic concentrations ranged from 0.25x to 4x MIC of the parental strain for the single antibiotics, and equally combined doses of 0.125x to 2x MIC both antibiotics (dose equivalents) in combination treatments.

Given the SCV phenotype appears as small aggregates or pinpoint growth in broth microdilution tests (Supplement Figure S1), 100 µL of 1:10 dilutions of bacteria from these wells as well as from growth controls were spread on blood agar plates (BD Biosciences, Heidelberg, Germany), and incubated overnight at 37 °C, 5% CO_2_. Different colony phenotypes were picked, labelled according to their phenotype (NCP or SCV), antibiotic selection (PEN or CRO) pressure and applied media (specification only for MH), and stored in 10% glycerol in water (vol/vol) at -80 °C. For downstream analyses, one representative colony per condition was selected to enable controlled comparison of phenotypic and genomic characteristics within a defined genetic background.

### Determination of the Minimal Inhibitory Concentration (MIC) for the variants

MICs were determined by broth microdilution in accordance with European Committee on Antimicrobial Susceptibility Testing (EUCAST) guidelines. Briefly, bacterial suspensions were prepared from overnight cultures and adjusted to an optical density at 600 nm (OD_600_) of 0.08 (turbidity equivalent to 0.5 McFarland standard), followed by 1:100 dilution in MH broth to achieve a final inoculum of approximately 5 × 10⁵ CFU/mL. Serial twofold dilutions of benzylpenicillin and ceftriaxone were prepared in 96-well microtiter plates, and the same volume of the diluted cultures was added. Plates were incubated at 37 °C overnight under 5% CO₂. MICs were defined as the lowest antibiotic concentration that completely inhibited visible bacterial growth. All measurements were performed in at least two independent biological experiments, each conducted in duplicate.

### Morphological characterization of SCVs

All cryo-conserved phenotypes were plated on blood agar and evaluated for colony color and size relative to the parental strain after 16 h of incubation at 37 °C under 5% CO₂. Colony morphology was documented by imaging colonies on a Standard Wolffhuegel counting grid (LMS Consult GmbH & Co. KG, Brigachtal, Germany), in which the smallest squares measure 3.33 mm × 3.33 mm. Colony size was quantitatively assessed by analyzing plate images using ImageJ software, and results are reported as mean ± standard deviation (Table 1). To assess morphological stability, the phenotypes were serially passaged for 30 consecutive days by transferring a single SCV colony daily onto a fresh blood agar plate. No liquid culture or intermediate resuspension steps were performed. After incubation for 16 h at 37 °C under 5% CO₂, a newly formed SCV colony was selected and streaked onto a fresh blood agar plate for the subsequent passage.

**Table 1:** Characteristics of small colony variants (SCV) and normal colony phenotypes (NCP) selected in our study

Label	MIC_PEN_ [mg/L]	MIC_CRO_ [mg/L]	Colony average size ± SD [mm] after 24 h of growth	Colony colour	Morphological stability for 30x passages	ØAbs_595_ per 10^8^ CFU ± SD	Auxotrophy for hemin, menadione and/or thymidine	MBC_PEN_ [mg/L]	MBC_CRO_ [mg/L]
NCP-0	1	4	1.3 ± 0.2	grey with translucent margin	stable	1.25 ± 0.17	none	2	> 64
SCV-PEN	1	512	0.5 ± 0.1	white	stable	0.20 ± 0.02	none	4	2048
NCP-CRO	0.5	2048	1.5 ± 0.2	white with translucent margin	stable	0.66 ± 0.43	none	1	4096
SCV-CRO	2	256	0.4 ± 0.03	white	stable	0.30 ± 0.04	none	16	4096
NCP-PEN + CRO	2	1024	1.2 ± 0.1	white with translucent margin	stable	1.00 ± 0.44	none	2	1024
SCV-PEN + CRO	2	4	0.3 ± 0.04	white	partly conversion back to NCP	0.10 ± 0.21	none	2	4
NCP-PEN + CRO-MH	2	512	1.0 ± 0.1	grey with translucent margin	stable	0.99 ± 0.59	none	2	512
SCV-PEN + CRO -MH	2	1024	0.4 ± 0.09	grey	stable	1.43 ± 0.83	none	2	1024

*Abs*_595_ absorption of crystal violet at 595 nm, *CFU* colony forming units, *CRO* Ceftriaxone, *MH* Mueller-Hinton broth, *MIC* Minimum inhibitory concentration, *MBC* Minimum bactericidal concentration that reduced bacterial count by 99% after 12 h, *PEN * Benzylpenicillin, *SD* Standard deviation

For microscopic characterization, bacterial cells were spread on glass slides, Gram-stained (BD, Sparks, USA) according to the manufacturer’s instructions, and qualitatively examined using a bright-field microscope (Axio Vert.A1, Zeiss, Jena, Germany).

### Quantitative crystal violet staining

Quantitative assessment of crystal violet binding was performed to estimate cell wall-associated staining modified to the method described previously [16]. Briefly, bacterial cultures (biological triplicates) were adjusted to an OD_600_ of 1.0 in saline (0.9% NaCl) and incubated with 0.1% crystal violet in water (wt/vol) for 1 min at room temperature with gentle agitation. Cells were pelleted by centrifugation (5 min, 4000 rpm) and washed three times with saline to remove unbound dye. The pellet was resuspended in 200 µL saline and 100 µL was serially diluted and plated on TH agar for CFU determination. The residual 100 µL suspension was centrifuged again, and the bound crystal violet was extracted with 100 µL of 30% acetic acid for 10 min. The absorption of supernatants was measured in triplicates at 595 nm using a spectrophotometer (Sunrise, Tecan, Switzerland), the absorption signal (Abs_595_) was normalized to the determined CFU/mL and expressed as Abs_595_ per 10^8^ CFU for more convenient visualization. NCP-0 was used as a global reference control, and additional post hoc comparisons were conducted between corresponding NCP and SCV pairs.

### Auxotrophy testing

Auxotrophy testing for menadione, thymidine and hemin was carried out using disk diffusion assays as described by Wellinghausen et al. for direct comparison of our and former SCVs [10]. Isolated SCVs and respective NCPs were adjusted to approximately 10^8^ CFU/mL in PBS and further diluted to 10^4^ CFU/mL in PBS. Bacterial solutions of both densities were plated onto TH, MH or M9 minimal medium agar plates. Commercially available hemin disks (X-factor disks, Sigma-Aldrich, St. Louis, USA) and blank disks impregnated with 20 µL of self-prepared 0.1 and 1 mg/mL menadione (vitamin K3) and thymidine (both Sigma-Aldrich, St. Louis, USA) solutions were placed on the agar surface. Disks impregnated with 1x PBS served as control. Auxotrophy was defined by the presence of enhanced growth surrounding supplemented disks or by phenotypic reversion of SCVs to the NCP after 24 h of incubation.

### Determination of acetate and lactate levels in culture supernatants

Assays were performed similarly to Wellinghausen et al. [10]. Briefly, bacterial liquid cultures with a starting OD_600_ of 0.02 were grown for 24 h at 37 °C in TH broth with the reaction tube cap loosely opened to allow for oxygen exchange. At designated time points (0 h, 4 h, 8 h and 24 h), 1 mL of the bacterial culture was centrifuged for 10 min at 21,000 g and 4 C. Supernatants (100 µL ) were stored at -20 C and used for lactate and acetate measurements. Acetate and lactate levels were measured with an Acetic Acid Assay Kit (Megazyme, Bray, Ireland) and Lactic Acid Kit (Sigma Aldrich, St. Louis, Missouri) according to the manufacturer’s protocol. Acetate concentrations were calculated with the provided Mega-Calc™ calculator. Experiments were performed in two independent biological experiments, each conducted in duplicate.

### Growth curves analysis

Bacterial liquid cultures of all phenotypes were adjusted to approximately 10^8^ CFU/mL in TH media. Equal volumes of all cultures were transferred to a microtiter plate in triplicates and the OD_600_ measured every 10 min for 18 h at a spectrophotometer (Sunrise, Tecan, Switzerland) to record growth curves. Growth curves metrics (lag time and growth rate) were calculated with the R package gcplyr (R version 4.4.1; gcplyr version 1.10.0) facilitating model-free and non-parametric growth curve data analyses [17]. In short, the growth rate was derived from the maximum of the per-capita derivative, while the lag time function projects the tangent line of the maximum derivative back to where it intersects the starting density.

### Time-kill assay

Bacterial strains were grown in TH broth to an OD_600_ of 0.2–0.5. The cultures were then adjusted to an OD_600_ of 0.08. Subsequently, 100 µL of the standardized bacterial suspension (corresponding to a starting inoculum of approximately 10^6^ CFU/mL) was added to each tube containing 9.9 mL TH broth supplemented with the respective concentrations of benzylpenicillin and ceftriaxone based on the individual MIC of each strain. A growth control without antibiotic served as the negative control.

Samples were collected at 0, 2, 4, 6, 8, 10, and 12 h after inoculation. At each time point, 50 µL of culture was removed, immediately serially diluted in TH medium, and plated on TH agar plates. CFU/mL was determined after overnight incubation at 37 °C. The experiment was performed in biological triplicates.

To quantify killing dynamics, the minimum duration required to reduce the viable bacterial population by 90% (MDK_90_) and 99% (MDK_99_) relative to the initial inoculum at 16× MIC was determined for each strain and treatment condition. In addition, the minimum bactericidal concentrations (MBC) required to achieve 99% reductions in bacterial counts after 12 h were assessed. The 12 h endpoint was chosen to correspond to the duration of the time-kill experiments and to facilitate comparison between concentration-dependent and time-dependent measures of antibiotic activity.

### Virulence testing in Galleria mellonella larvae

*G. mellonella* larvae (last-instar stage) were obtained from a commercial supplier (Bruno Mariani-FLOTEX, Augsburg, Germany). Only healthy larvae with a uniform cream-colored cuticle, high spontaneous motility, and no visible melanization or physical damage were included. Larvae were stored in the dark at 15 °C without feeding and acclimatized to room temperature for at least 30 min before infection. The average larval weight was 536.35 ± 72 mg. Virulence of NCPs and SCVs was compared using survival curves in the *G. mellonella* infection model [18]. Bacterial suspensions were grown for 1 h in TH broth, washed twice with 1x PBS and adjusted to increasing doses of bacteria from 10^5^ to 10^7^ CFU/larvae. The larvae (obtained from Bruno Mariani-FLOTEX, Augsburg, Germany) were injected with 10 µL of the bacterial suspension via a proleg using a Hamilton syringe (Hamilton Bonaduz AG, Bonaduz, Switzerland). Larvae were incubated at 37 °C, and survival was monitored at 1, 3, 5, 9, 17, 20, 24, 48 and 72 h post-infection. Survival experiments were performed in three independent infection experiments: in the first experiment five larvae per strain, in the second experiment five larvae per strain, but six larvae for NCP-0 and SCV-PEN, in the third experiment ten larvae per strain), resulting in a total of 20 or 21 larvae per strain. Kaplan-Meier survival curves were generated and statistical analysed (see Statistics). At 72 h post infection, or immediately after scoring if larvae had died earlier, hemolymph was collected from individual larvae (*N* = 10), serially diluted and plated on TH agar supplemented with 6 mg/L gentamicin as a selection marker [18]. Gentamicin was used as a selection marker to suppress contamination; due to the intrinsically low susceptibility of *E. faecalis* to aminoglycosides, resulting from limited uptake, an effect on SCV induction is considered minimal but cannot be excluded.

After overnight incubation, total CFU/mL per larva was determined, including both NCP and SCV phenotypes. The proportion of SCVs was additionally expressed as a percentage of the total CFU.

### DNA extraction, whole genome sequencing and variant calling

One bacterial colony of each phenotype was cultured overnight in TH broth and bacterial cells were harvested by centrifugation at 12,074 g for 10 min at room temperature. The pellets were treated with RNase, lysozyme, and proteinase K at 37 °C for 1 h, followed by DNA extraction according to the Qiagen Blood and Cell Culture DNA Maxi Kit (all Qiagen, Hilden, Germany). DNA concentration was quantified with a Qubit fluorometer using the Qubit™ dsDNA BR Assay kit (both Invitrogen, Carlsbad, CA). Sequencing libraries were prepared using the Native Barcoding Kit 24 V14 (SQK-NBD114.24, Oxford Nanopore Technologies) with prolonged incubation times. Sequencing was performed on the GridION system (Oxford Nanopore Technologies, Oxford, UK) at 4 kHz with 260bps on R10.4.1 flow cells, with a minimum DNA fragment length of 200 bp set in MinKNOW (v22.12.5). Basecalling and barcode demultiplexing were performed on the GridION using Guppy (v6.4.6) with the super accurate basecalling model (dna_r10.4.1_e8.2_260bps_sup@v3.5.2). Reads below 1,000 bp were excluded by Filtlong (v.0.2.1) (https://github.com/rrwick/Filtlong). *De novo* assembly was performed using Flye (--meta --nano-hq) (v2.9) [19]. Filtered reads were mapped to the assembly using Minimap2 (-ax map-ont) (v2.18) [20] and polished using Racon first (v1.4.20; github.com/lbcb-sci/racon), followed by Medaka (v1.5.0; github.com/nanoporetech/medaka) with the ‘dna_r10.4.1_e8.2_260bps_sup@v3.5.2’ model. Taxonomic classification and contamination control were performed with Sourmash (v4.8.5) [21]. Methylation-induced basecalling errors were masked using MPOA (v1.4.2; github.com/replikation/MPOA) and did not impact subsequent variant calling [22]. Pairwise variant calling was performed using snippy (v4.6.0) (https://github.com/tseemann/snippy), comparing the SCV reads to the previously assembled and Bakta-annotated (v1.9.3; https://github.com/oschwengers/bakta) wild strains.

### Statistics

Unless otherwise stated, visualisation and statistical analyses were performed using GraphPad Prism version 10 (GraphPad Software, San Diego, CA, USA). A *p*-value < 0.05 was considered statistically significant.

Analyses of growth kinetics as well as lactate and acetate concentrations were performed using non-parametric statistical testing. Statistical significance was assessed using the Kruskal–Wallis test followed by Dunn’s multiple comparisons test (95% confidence interval).

Larval survival was analyzed using Kaplan–Meier survival curves. Overall differences between groups were first assessed using the log-rank (Mantel–Cox) test. Where appropriate, pairwise comparisons were subsequently performed using the log-rank (Mantel–Cox) test, restricted to predefined biologically relevant contrasts, specifically between corresponding NCP and SCV variants at identical infection doses (CFU).

## Results

### Phenotypic and morphological variation of E. faecalis strain 26,786 under antibiotic stress

Cultures of the wild type *E. faecalis* strain 26,786 were systematically exposed to varying liquid concentrations of benzylpenicillin and ceftriaxone, both single and in combination, across different media. Subsequently, colony morphology was assessed on blood agar plates. The emergence of circular colonies of varying sizes and ranging from grey to white were observed. Morphologically distinct colonies were selected for further characterization. Notably, these phenotypic changes on agar plates coincided with the formation of small aggregates at the bottom of microtiter wells in liquid culture (Figure S1). Depicted colonies were categorized into NCP and SCV phenotypes and labelled according to the applied antibiotic(s): PEN, CRO or PEN + CRO, and culture medium (TH – without indication, or MH with indication) (Table 1). In the untreated control (NCP-0), SCVs did not emerge. Exposure to 2× MIC benzylpenicillin exclusively selected phenotypically different colonies with smaller size compared to NCP-0 that were therefore categorized as SCVs (SCV-PEN). Ceftriaxone monotherapy and combined benzylpenicillin–ceftriaxone treatment led to the emergence of both phenotypes. Representative colonies (NCP and SCV) were selected and subcultured on blood agar (Figure S2) to assess phenotypic stability. After 24 h, NCPs reached colony diameters of 1.0–1.5 mm, whereas SCVs remained markedly smaller (0.3–0.5 mm), corresponding to a 60–75% reduction relative to their respective NCP counterparts (Table 1). SCVs derived under benzylpenicillin or ceftriaxone monotherapy (SCV-PEN, SCV-CRO) consistently exhibited reduced colony size. Under combination therapy in TH broth, SCV-PEN + CRO formed the smallest colonies (0.3 ± 0.04 mm) and showed partial reversion during serial passaging, while selection in MH broth resulted in slightly larger colonies (0.4 ± 0.09 mm), indicating a medium-dependent effect on colony size.

All SCVs remained morphologically stable over 30 passages on blood agar and after cryo-conservation, except for SCV-PEN + CRO, in which approximately 15% of the population reverted to the NCP phenotype. Whole-genome sequencing confirmed the absence of contamination and verified that all phenotypes were taxonomically classified as *E. faecalis*.

No auxotrophy for hemin, menadione, or thymidine was detected in any phenotype. Analysis of crystal violet staining in aqueous solution, used to estimate cell-associated crystal violet binding, revealed no significant differences in absorption signals between corresponding SCVs and NCPs (Figure S3). A significant difference was only observed when comparing the NCP-0 to SCV-PEN (*p*-value 0.0226). Overall, crystal violet binding varied substantially among the different phenotypes and did not reveal a consistent trend associated with the SCV phenotype. Because CFU normalization was performed after brief crystal violet exposure, a minor influence of residual dye toxicity on bacterial recovery cannot be completely excluded despite the short incubation time and extensive washing steps. Therefore, these measurements should be interpreted as relative estimates of crystal violet binding rather than absolute measures of cell surface properties.

Bright-field microscopy of Gram-stained preparations showed apparent differences in the spatial distribution of bacterial cells between NCPs and SCVs (Figure S4). Whereas NCP variants frequently appeared as densely packed structures with large cell-free areas, SCV variants displayed a more homogeneous and dispersed distribution of cells. However, these images represent qualitative observations only and should be interpreted with caution, as differences in bacterial density resulting from the slower growth of SCVs may influence the microscopic appearance. Therefore, no firm conclusions regarding differences in aggregation behavior between NCPs and SCVs can be drawn from these observations.

### Antibiotic exposure drives increased ceftriaxone MICs across phenotypes

The analysis of MICs revealed pronounced and phenotype-specific alterations in β-lactam susceptibility among the SCVs compared with their corresponding NCPs, while also indicating a general increase in ceftriaxone MICs following β-lactam exposure across both phenotypes. For benzylpenicillin, MIC values remained largely stable across most variants. The parental strain (NCP-0) exhibited a MIC of 1 mg/L, which was retained in SCV-PEN. Similarly, MIC values in the combination-derived variants (NCP-PEN + CRO and SCV-PEN + CRO, as well as their MH counterparts) remained unchanged at 2 mg/L. Only SCV-CRO showed a moderate increase in MIC_PEN_ (2 mg/L) compared with its parental NCP-CRO (0.5 mg/L), indicating a limited reduction in benzylpenicillin susceptibility in this variant.

In contrast, ceftriaxone MICs displayed substantially higher MIC values, with a consistent increase observed after antibiotic selection irrespective of the selecting agent, but with variability depending on the selection pressure and a trend toward higher MIC_CRO_ in the NCPs (Table 1). An unexpected increase in ceftriaxone MIC was observed in SCV-PEN, where MIC_CRO_ rose from 4 mg/L in the parental strain (NCP-0) to 512 mg/L (> 100-fold), indicating that substantial ceftriaxone MIC elevation can occur even following benzylpenicillin selection. In the CRO-selected lineage, MIC_CRO_ remained high in both phenotypes but differed in magnitude, with SCV-CRO exhibiting a MIC_CRO_ of 256 mg/L compared to 2048 mg/L in NCP-CRO, suggesting a partial reduction in ceftriaxone resistance associated with the SCV phenotype. In the combination-derived variants, the effects were less consistent: SCV-PEN + CRO showed markedly lower MIC_CRO_ (4 mg/L) than its corresponding NCP (1024 mg/L), whereas SCV-PEN + CRO-MH displayed higher MIC_CRO_ (1024 mg/L vs. 512 mg/L in the corresponding NCP), indicating that the impact of the SCV phenotype on ceftriaxone susceptibility depends on the selection environment. However, as these observations are based on representative isolates, further analysis of additional clones will be required to determine whether these shifts in MICs represent a generalizable adaptation or isolate-specific variation.

### Delayed growth but minimal metabolic changes in SCVs

**Fig. 1:**
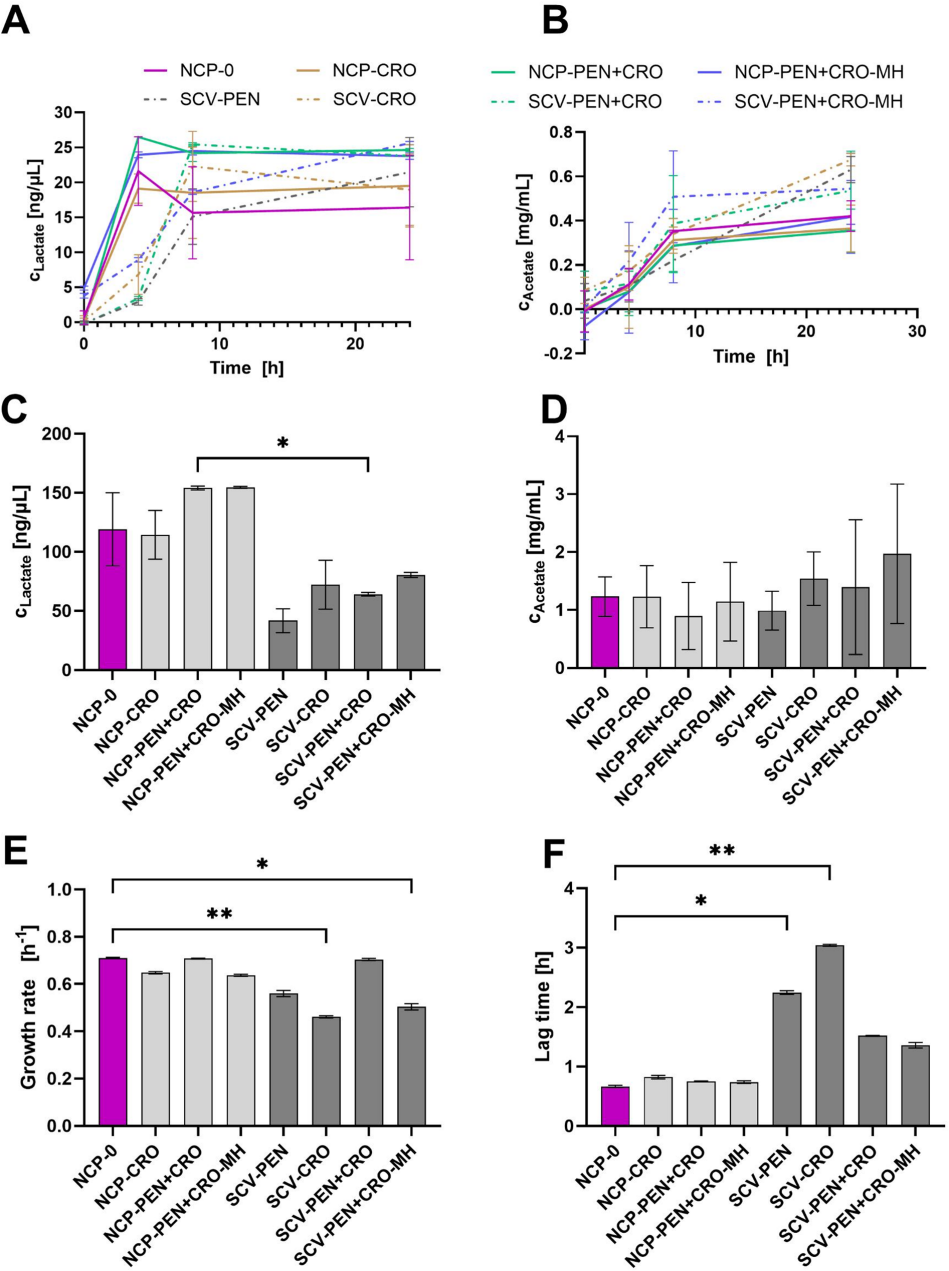
Metabolic and growth behaviour analysis of normal colony phenotypes. Kinetics of lactate (**A**) and acetate (**B**) production as concentrations in culture supernatants measured over a 24 h period for phenotypes derived from NCPs (solid lines) or SCVs (dashed lines): Legend for (A) and (B) above both diagrams. antibiotic treatment. Areas under the curves (AUC) for lactate (**C**) and acetate (**D**) production determined from starting time (zero) until 8 h of growth. Growth rates (**E**) and lag time (**F**) of the NCPs and SCVs. All measurements were performed at least in triplicates, means with standard deviations are presented. Colors denote (for C to F) NCP-0 (violet), NCP (light gray) and corresponding SCV (dark gray) phenotypes under the indicated conditions. Statistical analysis was performed using the Kruskal-Wallis test with Dunn’s post-hoc test. Significance (*p*-values) is presented as asterisks: *p* < 0.05 (*), *p* < 0.01 (**)

To investigate metabolic and growth-related differences between NCPs and SCVs, lactate and acetate concentrations as well as growth parameters were analyzed over time. Lactate production followed similar temporal patterns across all phenotypes, with a rapid increase during early growth phases (Fig. 1A, B). However, SCVs exhibited a delayed onset of lactate accumulation compared to their corresponding NCPs, consistent with slower initial growth. Acetate concentrations increased gradually over time in all strains, with largely comparable levels between NCPs and SCVs (Fig. 1C, D). While SCVs tended to show slightly higher acetate levels at later time points, these differences were minor and not indicative of a pronounced mMDK90 and MDK99 values for the indicated variantsetabolic shift.

Growth kinetics analysis revealed clear differences between phenotypes. SCVs exhibited substantially prolonged lag phases compared to NCPs (Fig. 1F), with lag times increased by approximately two- to threefold depending on the condition. This effect was most pronounced for SCV-CRO and SCV-PEN. In addition, SCVs showed moderately reduced growth rates relative to NCPs (Fig. 1E), although this effect was less consistent across conditions. Notably, SCV-PEN + CRO displayed growth rates comparable to its NCP counterpart. Statistically significant differences were primarily observed between NCP-0 and SCV-CRO for both lag time and growth rate.

Overall, these results indicate that the SCV phenotype is primarily characterized by altered growth dynamics, particularly extended lag phases, rather than major changes in extracellular metabolite production.

### Time-kill assays reveal heterogeneous tolerance profiles among *E. faecalis* variants

**Fig. 2:**
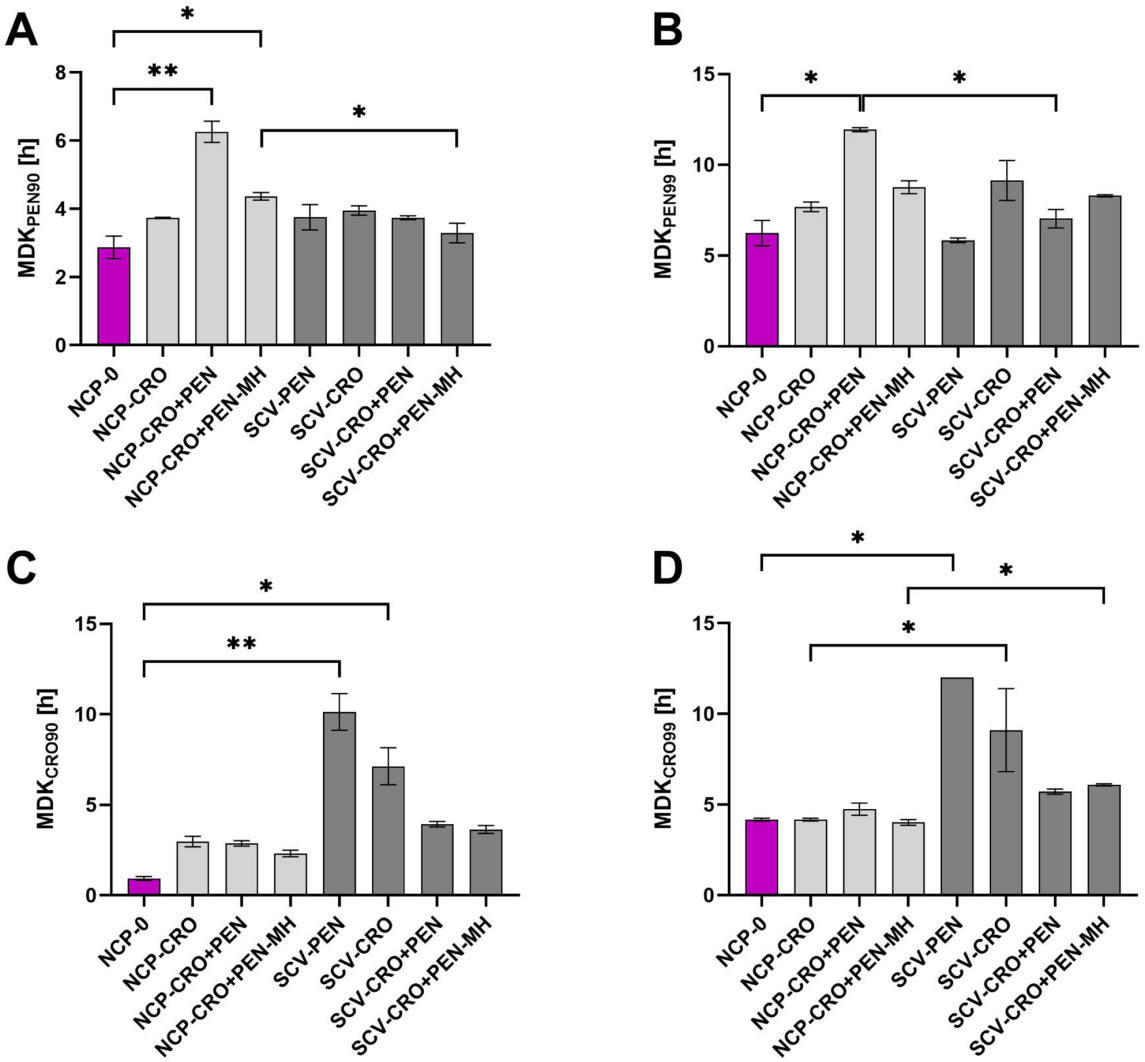
MDK90 and MDK99 values for the indicated variants, representing the time required to achieve 1-log₁₀ and 2-log₁₀ reductions in viable bacterial counts, respectively. **A** and **B** Treatment with 16 x MIC benzylpenicillin. **C** and **D** Treatment with 16 x MIC ceftriaxone. Data are shown as biological triplicates with mean ± SD. Colors denote NCP-0 (violet), NCP (light gray) and corresponding SCV (dark gray) phenotypes under the indicated conditions. Statistical analysis was performed using the Kruskal–Wallis test followed by Dunn’s multiple comparisons test. Significance (*p*-values) is presented as asterisks: *p* < 0.05 (*), *p* < 0.01 (**)

Time-kill assays were performed to assess antibiotic tolerance of the different variants under supra-MIC exposure to benzylpenicillin and ceftriaxone (Supplementary Figures S4–S7). To quantify killing dynamics, the minimum duration required to reduce the viable bacterial population by 90% (MDK_90_) and 99% (MDK_99_) was determined at 16x MIC for each strain and treatment condition (Fig. 2). The concentration of 16× MIC was selected because there were minor differences in killing kinetics at 8× and 16× MIC (Supplementary Figure S7), indicating that bactericidal activity had largely reached a plateau at these concentrations.

Under benzylpenicillin treatment (Fig. 2A, B), MDK_PEN_ values were generally similar across most variants, indicating only limited differences in tolerance. However, significant prolongation relative to the parental strain NCP-0 was observed for NCP-PEN + CRO at both the MDK_PEN90_ and MDK_PEN99_ level and for NCP-PEN + CRO-MH at the MDK_PEN99_ level. In addition, comparison of corresponding NCP/SCV pairs indicated significant differences for the CRO-selected lineage and the PEN + CRO-MH lineage, particularly at the level of MDK_PEN99_. These findings suggest that prolonged killing times under benzylpenicillin are not a general feature of the SCV phenotype but rather depend on the selective history of the isolate.

Under ceftriaxone treatment (Fig. 2C, D), the killing dynamics were more heterogeneous. Significant differences relative to NCP-0 were observed for SCV-PEN at both the MDK_CRO90_ and MDK_CRO99_ level and for SCV-CRO at the MDK_CRO99_ level. In pairwise comparisons, SCV-CRO and, to a lesser extent, SCV-PEN + CRO-MH showed prolonged MDK_CRO_ values compared with their corresponding NCPs, consistent with delayed killing under ceftriaxone exposure. By contrast, SCV-PEN + CRO did not show a consistent increase in tolerance relative to its matched NCP. Notably, although ceftriaxone MICs were broadly elevated after antibiotic selection, this was not uniformly accompanied by prolonged MDK_CRO_ values, indicating that reduced susceptibility and increased tolerance did not necessarily coincide.

Overall, the time-kill data demonstrate that tolerance profiles differed across lineages and treatment conditions. Some selected variants exhibited significantly prolonged MDK values, but these effects were not uniform across all SCVs or all NCP/SCV pairs. Thus, antibiotic tolerance in this strain collection appears to be context-dependent and shaped by both the selecting condition and the resulting phenotype, rather than being an intrinsic and universal property of SCVs.

### Changes in MBC values among SCV variants

To further assess bactericidal activity, MBCs were determined, revealing additional differences between SCVs and their parental phenotypes (Table 1). For benzylpenicillin, MBC values remained largely unchanged across most variants. The parental strain (NCP-0) exhibited an MBC_PEN_ of 2 mg/L, which increased only modestly in SCV-PEN (4 mg/L) and SCV-CRO (16 mg/L). In the combination-derived variants, MBC_PEN_ values remained stable at 2 mg/L across both NCP and SCV phenotypes, indicating preserved bactericidal activity despite phenotypic changes.

In contrast, MBC_CRO_ values were consistently high across all variants, reflecting the intrinsic low bactericidal activity of cephalosporins against *E. faecalis*. Notably, SCV-PEN showed a marked increase in MBC_CRO_ (2048 mg/L), whereas no MBC_CRO_ was reached for the parental strain NCP-0 within the tested range (16× MIC, corresponding to 64 mg/L), indicating a pronounced reduction in killing efficiency. Similarly, SCV-CRO and NCP-CRO both exhibited very high MBC values (4096 mg/L), indicating limited bactericidal activity regardless of phenotype. In the combination-derived variants, SCV-PEN + CRO showed a markedly reduced MBC_CRO_ (4 mg/L) compared with NCP-PEN + CRO (1024 mg/L), mirroring the MIC findings. In contrast, SCV-PEN + CRO-MH exhibited no change compared with its parental strain (1024 mg/L).

### Prolonged onset of infection response in SCVs in larvae

**Fig. 3:**
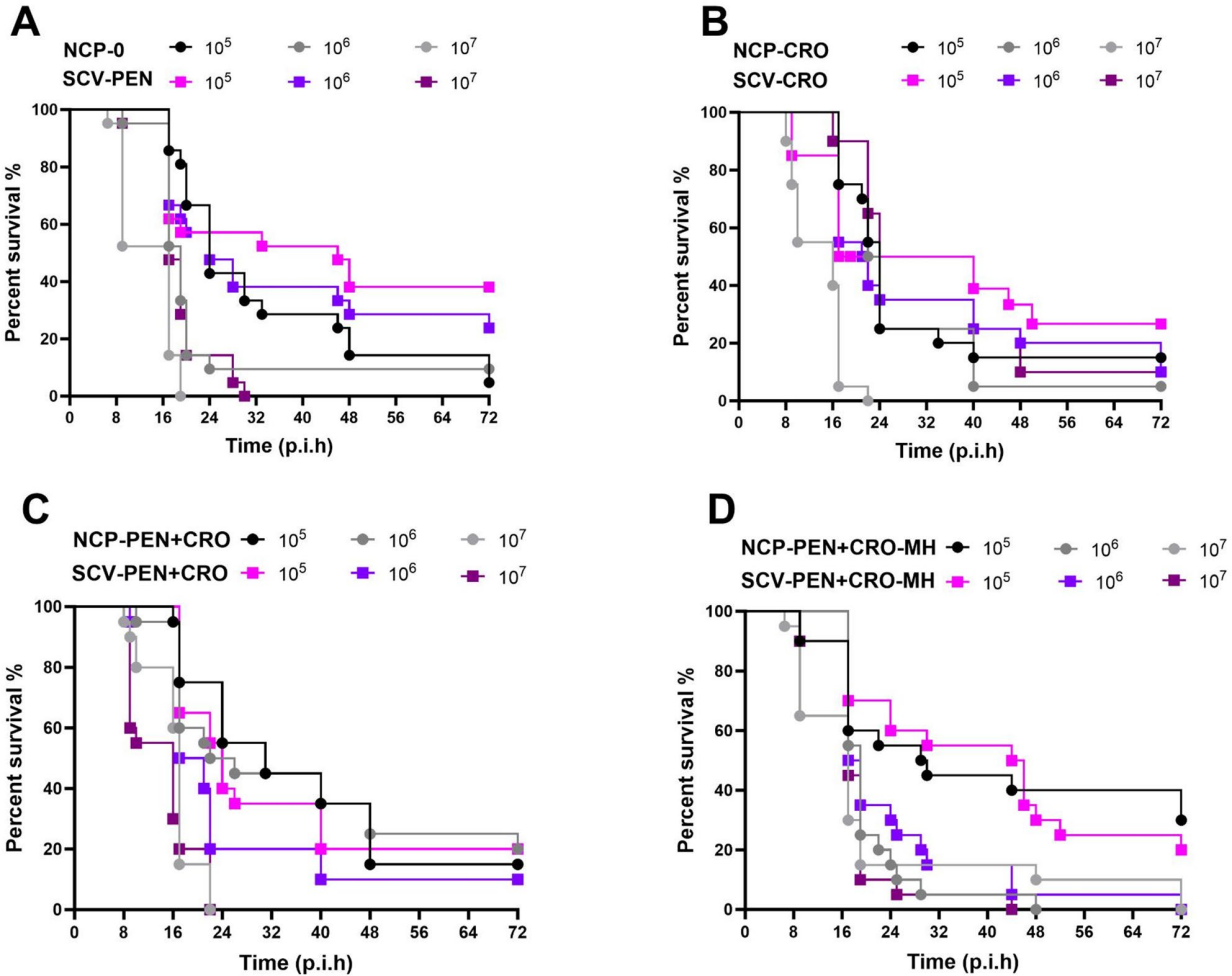
Kaplan–Meier survival curves of *G. mellonella* larvae following infection with NCP and SCV variants of *E. faecalis*. Survival curves after proleg injection with the indicated strains (**A** to **D** above the graphs) expressed as percent of survival against the time post infection in hours (p.i.h). Data represent pooled results from three independent experiments (*N* = 21 larvae in (**A**), *N* = 20 larvae in (**B**) to (**D**)). Statistical analysis was performed using the log-rank (Mantel–Cox) test, with pairwise comparisons between corresponding NCP and SCV variants, as well as to the NCP-0 as control group (*p*-values see Table S1)

To compare the in vivo pathogenic behavior of NCPs and SCVs, *G. mellonella* larvae were infected with increasing bacterial inocula and monitored over time for survival (Fig. 3). Analysis of matched NCP/SCV pairs across different inocula revealed a condition-dependent effect on infection kinetics. For the CRO-selected lineage (NCP-CRO/SCV-CRO), SCV infection resulted in a delayed onset of larval mortality compared with the corresponding NCP, reflected by a right-shift of the survival curves. This difference became more pronounced with increasing inoculum and reached statistical significance at 10^7^ CFU (*p* = 0.0001), whereas no significant differences were observed at lower doses (Table S1). Despite this temporal delay, survival curves converged at later time points, indicating that SCVs retained pathogenic potential but initiated lethal infection more slowly.

For the PEN + CRO lineage (NCP-PEN + CRO/SCV-PEN + CRO), survival kinetics were largely overlapping across all tested inocula, with no statistically significant differences between the phenotypes. A similar pattern was observed for the PEN + CRO-MH lineage (NCP-PEN + CRO-MH/SCV-PEN + CRO-MH), where survival curves remained comparable under all conditions.

Evaluation of the unpaired SCV-PEN variant suggested a tendency toward delayed infection kinetics. However, these observations were not consistently supported by statistical significance across conditions, indicating that such effects are variable and dependent on both phenotype and inoculum.

Comparison to the parental strain NCP-0 further highlighted the variability of these effects. A modest but statistically significant delay in mortality was observed for SCV-CRO at 10⁶ CFU (*p* = 0.0338), whereas all other variants, including both NCP- and SCV-derived lineages, did not differ significantly from NCP-0 (Table S1).

In addition to survival analysis, endpoint bacterial burden was quantified immediately after death or after 72 h post infection. Hemolymph-derived CFU counts (*N* = 10 larvae per variant) confirmed the presence of viable bacteria across all infection groups, reflecting the overall infection outcome per larva. The measured CFU/mL values represent the total bacterial population recovered from each larva, including both SCV and NCP phenotypes. Considering that the initial inoculum was administered per larva in a volume of 10 µL, corresponding to a 10³-fold higher concentration when normalized to mL, the recovered bacterial loads indicate an increase of at least three orders of magnitude during infection (Figure S8). While variability between individual larvae was observed, no consistent reduction in bacterial burden was associated with SCV infection compared to the corresponding NCP variants. In several cases, comparable CFU levels were detected despite differences in infection kinetics, indicating that delayed mortality did not necessarily correspond to reduced bacterial burden.

### Limited in vivo stability of the SCVs

In addition to survival analysis, endpoint bacterial burden was quantified (CFU per larva, *N* = 10 per variant), providing a quantitative measure of infection outcome. To assess phenotypic stability in vivo, hemolymph recovered from infected larvae was plated and the proportion of SCV colonies relative to total CFU was determined. Across most SCV variants, only a small fraction of recovered bacteria retained the SCV phenotype, indicating substantial reversion to the NCP during infection (Fig. S9). Overall, the proportion of SCV colonies remained below ~ 10% of the recovered population. While the lowest frequencies were observed for SCV-PEN and somewhat higher frequencies for SCV-PEN + CRO-MH, these differences were highly variable and did not indicate consistent differences between the SCV backgrounds.

Notably, SCV colonies were also detected in individual larvae infected with NCP variants, demonstrating that SCVs can arise in vivo during infection (Table S2). However, their frequency remained low, typically below ~ 1–2% of the total population across all NCP backgrounds. This indicates that while host-associated conditions can promote the emergence of SCVs, their formation occurs at low frequency and does not result in stable enrichment of the phenotype.

Interestingly, the only factor consistently associated with higher proportions of recovered SCVs was a higher bacterial burden per larva, suggesting that the absolute number of bacteria rather than the underlying SCV background may be the main determinant of SCV recovery in this model. Overall, these findings indicate that SCVs represent a highly dynamic subpopulation characterized by limited phenotypic stability and low-frequency de novo formation during infection.

### Identification of genetic mutations in regulatory and stress response pathways in SCVs

To analyse whether the SCV phenotype arises from genetic alterations, we performed nanopore sequencing followed by variant calling for each SCV phenotype and the ancestral NCP. Nanopore sequencing achieved a mean depth of 166× and mean read length of 10 kb across all variants. Genome reconstruction and subsequent polishing resulted in circular 2.73 Mbp long assembled genomes for each variant. Variant calling identified only one to two genetic alterations when comparing the SCVs to its NCP ancestor. Those included single nucleotide polymorphisms (SNP) and deletions, and in one case an insertion (Table 2). The mutations detected varied across the different SCV phenotypes, suggesting that each exhibited distinct genetic changes. However, these mutations affected in part similar regulatory and stress response pathways.

**Table 2:** Variant calling for the SCVs versus the parental strain (NCP-0)

Phenotype comparison	Type of mutation	Variation	Amino acid position	Gene name	Annotation/ function	Effect on protein /amino acid change
SCV-PEN	INS	T -> TC	195/194	*yvbJ*	Uncharacterized membrane protein YvbJ in Gram-positive bacteria, contains N-terminal Zn ribbon domain	Frameshift variant resulting in loss of stop codon and elongated transcript
	SNP	G -> T	225/249	*ireP*	Family PP2C-type Ser/Thr phosphatase from the IreK/IreP phosphorylation regulatory pair	Missense variant Ala225Glu
SCV-CRO	DEL	CT -> C	35/249	*tyrS*	Tyrosine-tRNA ligase	Frameshift variant resulting in loss of function
SCV-PEN + CRO	SNP	T -> A	123/306	*pstC*	Phosphate ABC transporter permease subunit PstC	Premature stop codon after Leu123* resulting in shortened polypeptide
	SNP	A -> C	233/489	*arlS*	Signal transduction histidine kinase from the ArlS/ArlR two-component regulatory system	Missense variant Tyr233Asp
SCV-PEN + CRO-MH	DEL	GCAA -> G	172/269	*pstB*	Phosphate ABC transporter ATP-binding protein PstB	Disruptive in-frame deletion of Gln172

*INS* Insertion, *SNP* Single nucleotide polymorphism, *DEL* Deletion, * = stop codon

In SCV-PEN, a frameshift mutation resulted in the loss of a stop codon, leading to an elongated transcript of the uncharacterized membrane protein YvbJ. This elongation likely causes incorrect folding and a consequent loss of function. Although the exact function of YvbJ remains unknown, it contains a zinc ribbon domain — a structural motif commonly associated with DNA binding or protein–protein interactions — suggesting it may play a role in one of these processes. In addition to the mutation in *yvbJ*, a SNP in the serine/threonine phosphatase *ireP* was observed in SCV-PEN. IreK and IreP form a key serine/threonine phosphorylation regulatory pair in *E. faecalis* and play a vital role in sensing environmental changes and adapting cellular processes accordingly. IreK is a eukaryotic-like Ser/Thr protein kinase that senses cell envelope stress — especially from β-lactam antibiotics — via its PASTA domain. It autophosphorylates and activates downstream targets involved in peptidoglycan synthesis, stress tolerance, and antibiotic resistance, making it essential for cephalosporin resistance in *E. faecalis*. IreP counteracts IreK by dephosphorylating its targets, maintaining phosphorylation balance to prevent excessive stress responses. Its disruption alters antibiotic susceptibility, highlighting its role in regulatory homeostasis [23, 24].

In addition to serine/threonine kinase/phosphatase systems, phosphoregulation is mediated via two-component systems consisting of histidine kinase sensors and associated response regulators. Interestingly, a SNP impacting ArlS histidine kinase function was found in the SCV-PEN + CRO phenotype (Table 2), indicating that two SCV phenotypes developed mutations in phosphoregulation pathways independently from each other. Further mutations affecting phosphate uptake were detected in two SCV phenotypes having been selected under the same antibiotic pressure but different culture media (SCV-PEN + CRO in TH and SCV-PEN + CRO-MH in MH media). Both the permease subunit PstC of the phosphate ABC transporter, which facilitates phosphate uptake into the cell (SCV-PEN + CRO), and the ATP-binding protein PstB, which drives phosphate transport by hydrolyzing ATP (SCV-PEN + CRO-MH), were impacted. A deletion in the gene encoding tyrosine-tRNA ligase (tyrosyl-tRNA synthetase) was identified in SCV-CRO, disrupting its role in attaching tyrosine to tRNA, potentially leading to the accumulation of uncharged tRNAs.

## Discussion

SCVs are increasingly recognized as contributors to chronic and relapsing infections because they can survive antimicrobial exposure and re-establish growth after treatment. Although SCVs and their subtypes have been extensively studied in *S.* aureus, their role in enterococcal infections remains poorly defined [2, 4, 25–28]. In the present study, β-lactam exposure selected multiple SCV phenotypes of *E. faecalis* that fulfilled the classical morphological criterion of reduced colony size relative to the parental strain, yet differed markedly in growth behaviour, metabolic activity, in vivo stability, and tolerance profiles. In this study, we distinguish between morphologically stable SCVs, which maintain their phenotype upon passaging, and non-stable SCVs, which exhibit phenotypic reversion or delayed growth-associated variability. These findings indicate that SCVs in *E. faecalis* represent a heterogeneous adaptive state rather than a single uniform phenotype.

Notably, antibiotic exposure was associated with a marked increase in ceftriaxone MICs across multiple lineages, including variants selected under benzylpenicillin pressure, indicating that reduced susceptibility can arise independently of the selecting antibiotic. Similar observations have been reported for *E. faecalis*, where β-lactam exposure can induce adaptive changes affecting penicillin-binding proteins and regulatory pathways, thereby altering cephalosporin susceptibility [29]. Importantly, these MIC shifts were observed not only in SCV phenotypes but also in corresponding NCP variants, indicating that increased MICs represent a general adaptive response to β-lactam exposure rather than a feature specific to SCVs. This is consistent with broader observations in Gram-positive pathogens, including *S. aureus*, where antibiotic exposure can select for subpopulations with altered susceptibility independent of classical resistance mechanisms [5, 6]. However, the magnitude of these changes was highly variable and, in some cases, extreme, such as the > 100-fold increase in MIC_CRO_ observed in SCV-PEN, highlighting the plasticity of β-lactam susceptibility in enterococci. Such pronounced MIC shifts have also recently been reported under clinical conditions in *E. faecalis* infective endocarditis, where dual β-lactam therapy with benzylpenicillin and ceftriaxone was associated with increases in ceftriaxone MICs from initial values of 4–8 mg/L to ≥ 256–1024 mg/L during treatment, underscoring the clinical relevance of this adaptive plasticity [30]. Importantly, these findings show that MIC increases do not directly predict bactericidal activity or tolerance, as reflected by the dissociation between MIC, MDK, and MBC across variants. Together, this indicates that SCV formation, MIC shifts, and antibiotic tolerance represent partially independent phenomena, and that SCV formation alone is not a reliable indicator of antibiotic tolerance.

In contrast to MIC, which reflects growth inhibition, killing-based parameters derived from time-kill experiments revealed a more heterogeneous pattern. MDK analyses showed that only SCV-CRO and to a lesser extent SCV-PEN + CRO-MH, exhibited prolonged killing times, whereas other SCVs did not differ substantially from their corresponding NCPs. This is in line with the concept that antibiotic tolerance, defined by survival during exposure, is phenotypically heterogeneous even within clonal populations [5]. MBC values determined under comparable conditions further supported this observation. For benzylpenicillin, MBCs remained largely stable across variants, consistent with the limited differences observed in MDK values. For ceftriaxone, however, MBC values were generally high across most variants, reflecting the intrinsically low bactericidal activity of cephalosporins against enterococci, as previously described [13]. Importantly, neither MDK nor MBC changes followed a consistent pattern across SCVs, supporting observations from *S. aureus* and other Gram-positive bacteria that tolerance and killing dynamics are not intrinsic properties of SCVs per se but depend on the underlying physiological state [25–28].

When integrating MIC, MDK, and MBC data, it becomes evident that these parameters are only partially linked and can diverge substantially. In particular, MIC increases did not consistently translate into prolonged MDK values or higher MBCs, supporting the conceptual distinction between resistance and tolerance [5]. Moreover, because MDK and MBC were determined at concentrations scaled to the individual MICs (e.g., 16 x MIC), large MIC shifts resulted in very high absolute antibiotic concentrations for some variants. For example, highly elevated MICCRO values correspond to ceftriaxone concentrations of up to 32.8 g/L in the killing assays, far exceeding clinically achievable levels (Table 1). This is an important limitation also highlighted in tolerance studies in *S. aureus*, where high relative concentrations can mask physiologically relevant killing dynamics [26, 27]. Consequently, prolonged MDK (mainly for ceftriaxone) or high MBC values under these conditions may partly reflect experimental scaling rather than clinically meaningful tolerance. Conversely, some variants with low MICs and low MBCs did not exhibit prolonged MDK, further underscoring that bactericidal activity and killing kinetics are not solely determined by susceptibility. Together, these findings reinforce that resistance (MIC), tolerance (MDK), and bactericidal thresholds (MBC) represent distinct but interacting dimensions of the antibiotic response that must be interpreted in the context of both relative and absolute drug concentrations.

A central finding of the study is that prolonged lag phase was not uniformly associated with increased antibiotic tolerance. Time-kill analyses showed that only selected subtypes, particularly SCV-CRO and, to a lesser extent, SCV-PEN + CRO-MH, displayed prolonged MDK_CRO90_ and especially MDK_CRO99_ values compared with their corresponding NCPs, whereas SCV-PEN and SCV-PEN + CRO did not show a consistent increase in tolerance. Moreover, comparison to the parental strain NCP-0 revealed only selective differences in MDK values, further supporting that increased tolerance is not a universal feature of SCVs in this model. This distinction is important because tolerance is defined by survival during antibiotic exposure and is best quantified by killing-based metrics such as MDK rather than inferred from colony morphology or growth delay alone [5]. Our data therefore support a model in which extended lag time can contribute to tolerance-by-lag, but only in specific SCV subtypes and not as a universal property of all small-colony phenotypes.

Our findings partly agree with the previous detailed characterization of *E. faecalis* SCVs by Wellinghausen et al., who described a clinical SCV isolate from persistent endocarditis with reduced colony size, prolonged lag phase, abnormal cellular morphology, and hemin auxotrophy [10]. Similarly, in this study SCVs displayed reduced colony size and delayed growth. In contrast, none of the phenotypes in the present study showed auxotrophy for hemin, menadione, or thymidine, indicating that β-lactam-induced SCVs in *E. faecalis* are not restricted to the classical auxotrophic forms known from *S. aureus*.

Our microscopy observations revealed differences in the spatial distribution of bacterial cells between NCPs and SCVs. However, because these images are qualitative and differences in cell density may result from the slower growth of SCVs, no firm conclusions regarding aggregation behaviour or structural alterations can be drawn from these observations. Likewise, crystal violet binding did not differ substantially between phenotypes and showed considerable variability. In addition, because CFU normalization was performed after brief exposure to crystal violet, a minor influence of residual dye toxicity cannot be completely excluded despite extensive washing. Therefore, these assays should be regarded as exploratory and do not support detailed mechanistic conclusions. Further studies employing dedicated structural methods, such as transmission electron microscopy, would be required to assess possible differences in cell envelope architecture.

The observed heterogeneity is also relevant in the context of non-stable SCVs. In *S. aureus*, prolonged lag time has been linked to non-stable SCVs, which are characterized by delayed colony appearance, transient antibiotic tolerance, and phenotypic reversion after renewed growth [26–28]. Several phenotypes in our study are consistent with this concept: they exhibited delayed growth in vitro, delayed infection kinetics in vivo, and substantial reversion to the NCP after host passage. Importantly, our in vivo data further demonstrate that SCVs not only revert but can also arise de novo during infection, albeit at low frequency (< 1–2% of the recovered population across NCP backgrounds). At the same time, not all phenotypes behaved similarly. SCV-PEN + CRO-MH remained comparatively stable both during passaging and after recovery from infected larvae, indicating that *E. faecalis* can generate both more stable and more reversible SCV states. Because colony appearance time was not monitored continuously, we cannot formally distinguish between true slow-growth SCVs and delayed-appearance variants as described in scanner-based analyses for *S. aureus* [27]. The classification of some phenotypes as non-stable SCVs should therefore be considered provisional.

The *G. mellonella* experiments further support the interpretation that SCVs primarily differ in infection kinetics rather than overall virulence. A delayed onset of larval mortality was observed only in specific lineages and under defined conditions, most prominently for the CRO-selected pair at high inoculum, whereas other SCV/NCP pairs showed largely overlapping survival curves. In addition, comparisons to the parental strain NCP-0 revealed only limited and inconsistent differences. Despite these variations, survival outcomes converged at later time points across all variants, indicating that SCVs retained pathogenic potential but differed mainly in the timing of disease progression. Consistent with this, endpoint bacterial burden analysis revealed comparable CFU levels across SCV and corresponding NCP variants despite differences in infection kinetics, indicating that differences in mortality kinetics were not necessarily accompanied by corresponding differences in bacterial burden at the experimental endpoint. This observation is in line with the in vitro findings, where prolonged lag phases and heterogeneous MDK values influenced killing dynamics without uniformly affecting overall bacterial survival. Plating of hemolymph further demonstrated that most SCVs reverted substantially to the NCP in vivo, suggesting that delayed infection kinetics are followed by phenotypic switching and renewed proliferation of the parental-like state.

The metabolic data are in line with this interpretation. Delayed lactate accumulation in SCVs indicates postponed metabolic activation during early growth, whereas acetate concentrations did not differ consistently between NCPs and SCVs. Thus, the most robust metabolic signal in our dataset was not a stable rewiring of fermentation, but a temporal delay in metabolic output. This finding fits well with the prolonged lag phases and delayed killing kinetics observed in selected SCVs. Rather than representing constitutively slow-growing cells, these phenotypes appear to remain longer in a low-activity state before resuming active growth. This interpretation is also more consistent with the in vivo reversion data than the assumption of a permanently attenuated phenotype. Because lactate is a major end product of carbohydrate metabolism in *E. faecalis*, delayed lactate production most likely reflects postponed entry into active growth rather than a distinct endpoint metabolic program [31].

Whole-genome sequencing identified one to two mutations in each SCV phenotype, affecting genes involved in phosphoregulation, phosphate uptake, and aminoacyl-tRNA charging. These findings suggest that multiple genetic routes can converge on a phenotype of delayed growth resumption. In SCV-PEN, the mutation in *ireP* is particularly notable because IreP is the cognate phosphatase of the PASTA kinase IreK, a key regulator of cell envelope stress responses and intrinsic cephalosporin resistance in *E. faecalis* [23, 24, 32]. Likewise, the *arlS* mutation in SCV-PEN + CRO points to a possible role of altered two-component signaling in SCV formation. In SCV-PEN + CRO and SCV-PEN + CRO-MH, mutations in *pstC* and *pstB* suggest that disturbed phosphate transport may contribute to growth adaptation under stress, which is plausible in light of recent work linking phosphate transport defects to small-colony phenotypes in experimentally evolved *E. faecalis* [33]. Finally, the frameshift in *tyrS* in SCV-CRO is mechanistically intriguing, as impaired aminoacyl-tRNA synthetase activity could increase the pool of uncharged tRNAs and thereby promote stringent-response-associated growth arrest, a mechanism linked to persistence in other bacteria [34, 35]. This is consistent with the pronounced lag phase and strongest tolerance phenotype observed in SCV-CRO. Nevertheless, these interpretations remain putative and require functional validation.

Taken together, these findings suggest that phosphoregulation and nutrient stress signaling represent central nodes in the emergence of lag-associated SCVs in *E. faecalis*, while the observed heterogeneity supports the concept of SCVs as a convergent adaptive state arising through multiple genetic and physiological pathways.

Several limitations should nevertheless be considered. First, the study focused on a limited number of representative colonies derived from antibiotic-exposed populations. While this reductionist approach enabled controlled phenotypic, in vivo, and genomic comparisons within a defined genetic background, it does not capture the full diversity of adaptive responses and limits generalizability. The identified mutations and phenotypes should therefore be interpreted as representative examples rather than universally conserved features. Second, genomic and phenotypic analyses were based on single colonies per condition, which may introduce selection bias. However, because all analyses were performed using isogenic derivatives obtained from the same parental strain, this design minimized the influence of unrelated strain-specific differences and allowed observed phenotypic changes to be attributed more directly to antibiotic selection and SCV formation rather than to inter-clonal variability. Third, the *G. mellonella* model did not include antibiotic treatment, precluding direct assessment of tolerance under clinically relevant conditions. While such experiments would provide additional translational insight, their interpretation in this model is complicated by pharmacokinetic constraints and dosing variability. In addition, although gentamicin was used as a selection marker, a potential influence on SCV formation cannot be fully excluded, even though this effect is expected to be limited in enterococci.

Despite these limitations, the integrative approach combining phenotypic, metabolic, in vivo, and genomic analyses enabled discrimination between reduced colony size, delayed growth, delayed infection onset, phenotypic reversibility, and antibiotic tolerance. This study therefore provides a foundation for future investigations addressing SCV diversity and clinical relevance across a broader range of isolates. Together, these findings indicate that SCVs in *E. faecalis* represent a dynamic and context-dependent adaptive state rather than a uniform tolerance mechanism, with important implications for persistence and treatment response in enterococcal infections.

## Supplementary Information

Supplementary Material 1.

## Data Availability

All data supporting the findings of this study are included in this published article and its supplementary information files. Sequencing data is available at the NCBI BioProject database ( https://www.ncbi.nlm.nih.gov/bioproject/PRJNA1491000 ) under accession number PRJNA1491000.
