# Supplementary material for: Extended lag phases and variable antibiotic tolerance in β-lactam-induced small colony variants of Enterococcus faecalis
Source: BMC Microbiol. 2026 Jul 17;26:647. doi: 10.1186/s12866-026-05403-y (PMC13377840; doi:10.1186/s12866-026-05403-y)
Supplement: Supplementary file 1 — Supplementary Material 1. [file 12866_2026_5403_MOESM1_ESM.pdf]

Supplementary data

Extended Lag Phases in *Enterococcus faecalis* Small Colony Variants as Adaptation to β-Lactam Exposure

Authors: Kamran A. Mirza[1,2a], Lara Thieme[1,2a#], Nicole Enslinger[1], Mehri Azimi[3], Mara Lohde[1], Mateusz Jundzill[1,2], Christian Brandt[1,4], Mathias W. Pletz[1,2], Oliwia Makarewicz[1,2*]

Affiliations:
[1]Jena University Hospital, Friedrich-Schiller-University Jena, Institute for Infectious Diseases and Infection Control, Am Klinikum 1, 07747 Jena, Germany
[2]Leibniz Center for Photonics in Infection Research, 07743 Jena, Germany
[3] Department Cellular and Molecular Medicine, Department of Medicine, University of Ottawa, Ottawa, ON K1N 9A9, Canada
[4]InfectoGnostics Research Campus, 07743 Jena, Germany
[a] Both authors contributed equally to the manuscript
[#] Currently working at Dynamics42 GmbH, 07745 Jena.

Corresponding author: Oliwia.makarewicz@med.uni-jena.de

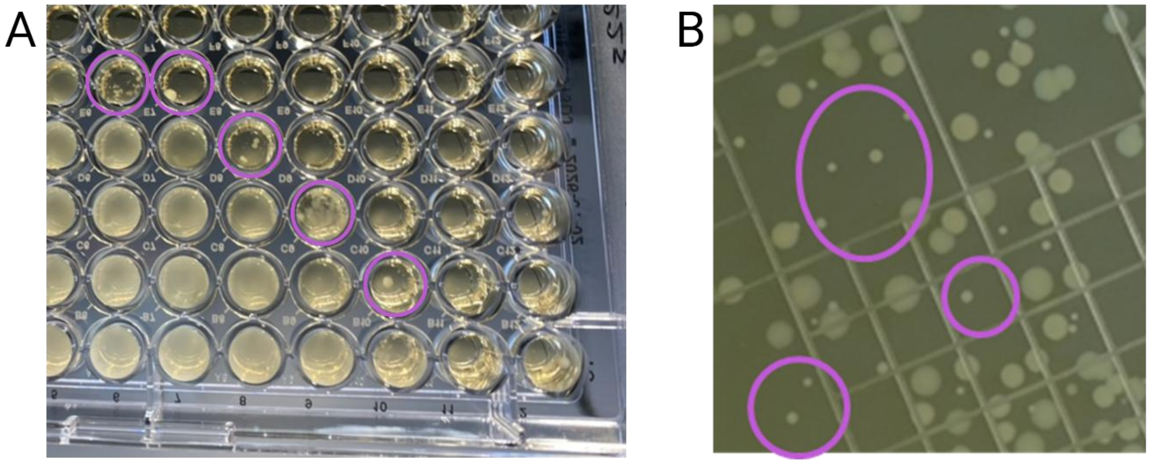

Figure S1: A) Representative image of the bottom of a microtiter plate following overnight exposure of *E. faecalis* 26786 to penicillin and ceftriaxone. Pinpoint growth (highlighted by pink circles) is visible as bacterial aggregates at the bottom of wells, corresponding to trailing MIC endpoints. B) Representative colonies of NCPs and SCVs recovered from wells exhibiting pinpoint growth and plated on MH agar. The size of the smallest squares of the Standard Wolffhügel counting grid is 3.33 mm × 3.33 mm.

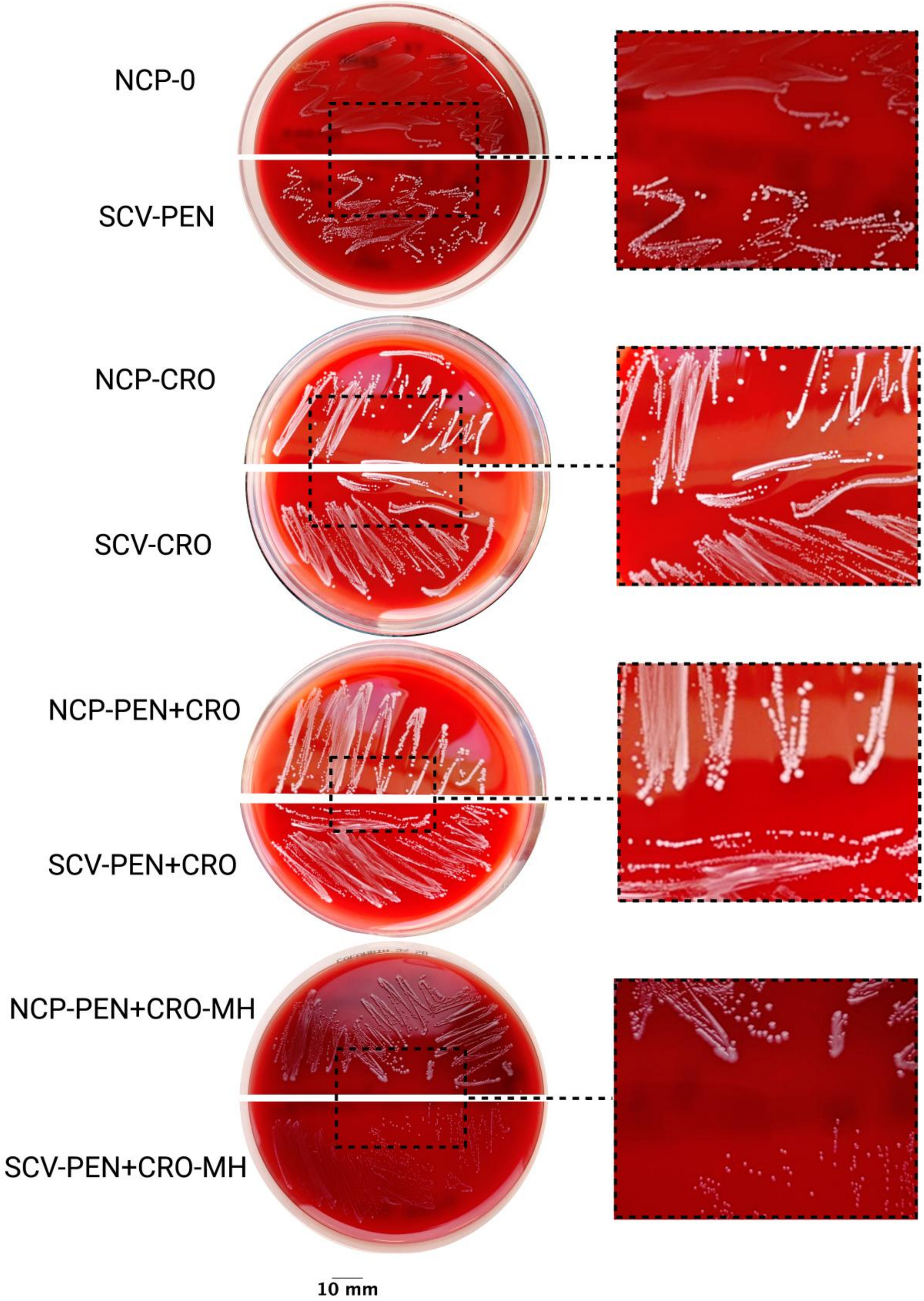

Figure S2: Representative blood agar plate images of all described phenotypes. See Table 1 for labelling of the phenotypes.

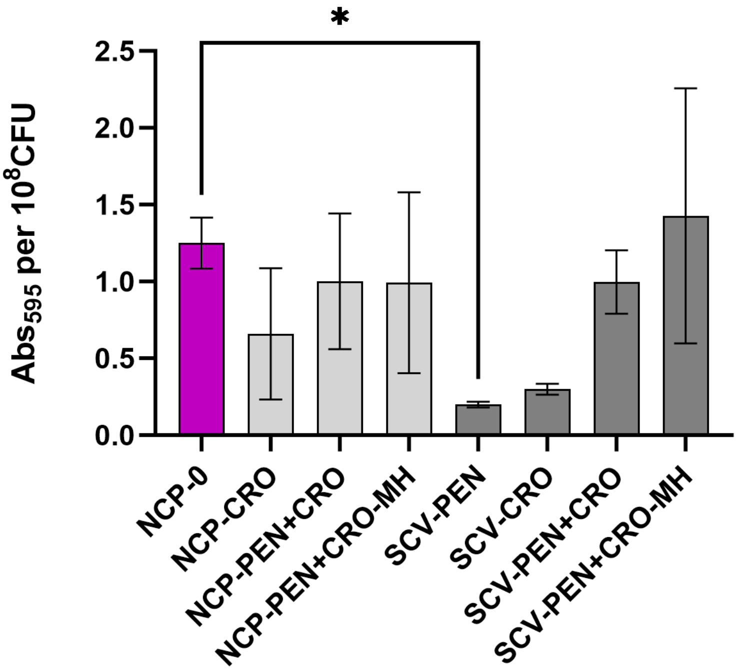

Figure S3: Quantitative assessment of crystal violet binding to the cell envelope, expressed as absorbance normalized to 10^8 CFU/mL, for NCP and SCV phenotypes (biological triplicates) derived under different selective conditions. Statistical significance was assessed using the Kruskal–Wallis test followed by Dunn's multiple comparisons test (95% confidence interval). $p < 0.05$ (*).

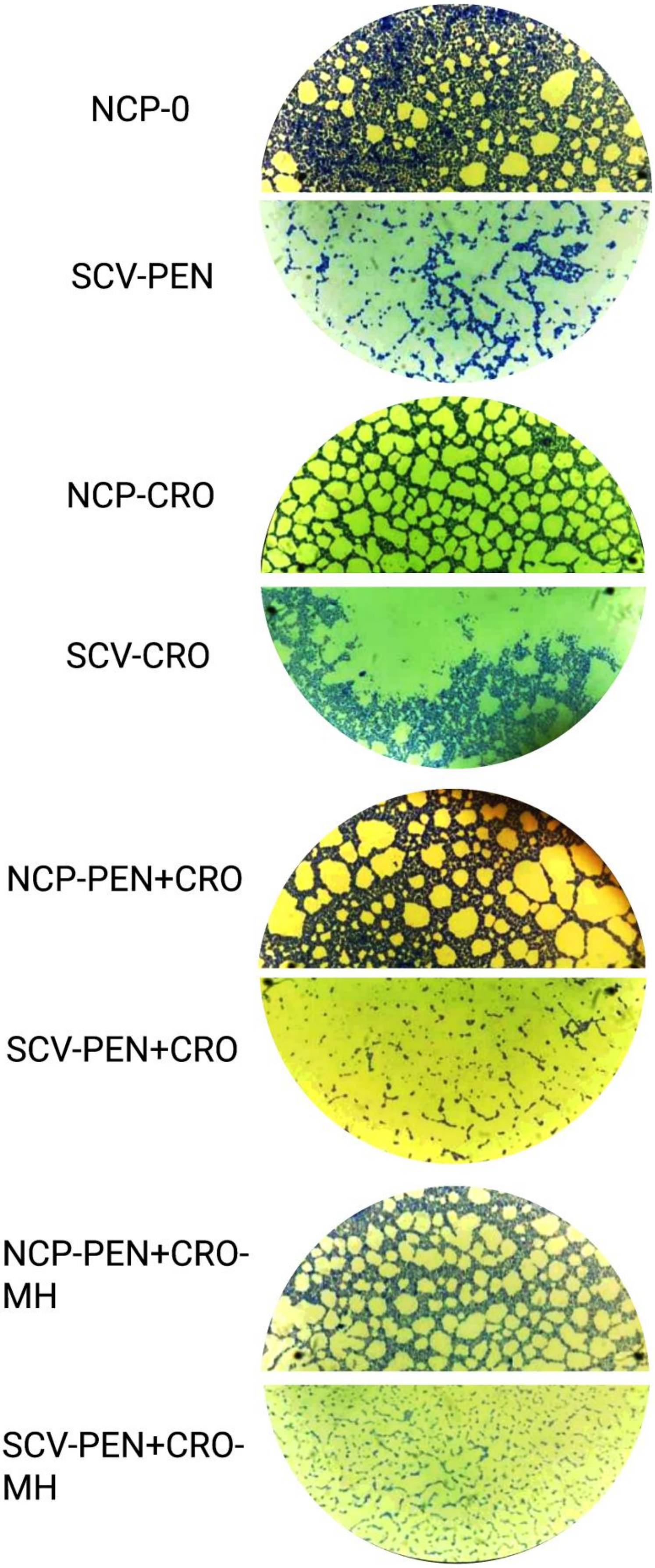

Figure S3: Representative images of Gram-stained phenotypes at 40x magnification.

Table S1 Statistical analysis of the larval survival curves. Testing for significant differences between the survival curves was performed using the log-rank (Mantel-Cox) test, with a $p < 0.05$ being considered statistically significant.

**Group comparison**	**Bacterial dose [CFU/larvae]**	***p* value**
NCP-CRO vs. SCV-CRO	1x10^5	0.5925
	1x10^6	0.7031
	1x10^7	0.0001
NCP-PEN+CRO vs. SCV-PEN+CRO	1x10^5	0.6376
	1x10^6	0.1728
	1x10^7	0.3028
NCP-PEN+CRO-MH vs. SCV-PEN+CRO-MH	1x10^5	0.8110
	1x10^6	0.3664
	1x10^7	0.8103
NCP-0 vs. SCV-CRO	1x10^6	0.0338
NCP-0 vs. NCP-PEN+CRO	1x10^6	0.0662
NCP-0 vs. SCV-PEN+CRO	1x10^6	0.3399
NCP-0 vs. NCP-PEN+CRO-MH	1x10^6	0.7971
NCP-0 vs. SCV- PEN+CRO-MH	1x10^6	0.8786

Table S2 Proportion of SCV among the total CFU recovered from larval hemolymph after infection with the NCP phenotypes.

**Name**	**Bacterial load**	**Larvae number**	**SCV in %**
NCP-0	10^5	1	2.7
	10^6	1	0.75
NCP-CRO	10^5	1	0.38
	10^6	1	0.69
		2	1.08
NCP-PEN+CRO	10^6	1	0.43
		2	2.76
	10^7	1	1.48
		2	0.51
		3	0.48
NCP-PEN+CRO-MH			none

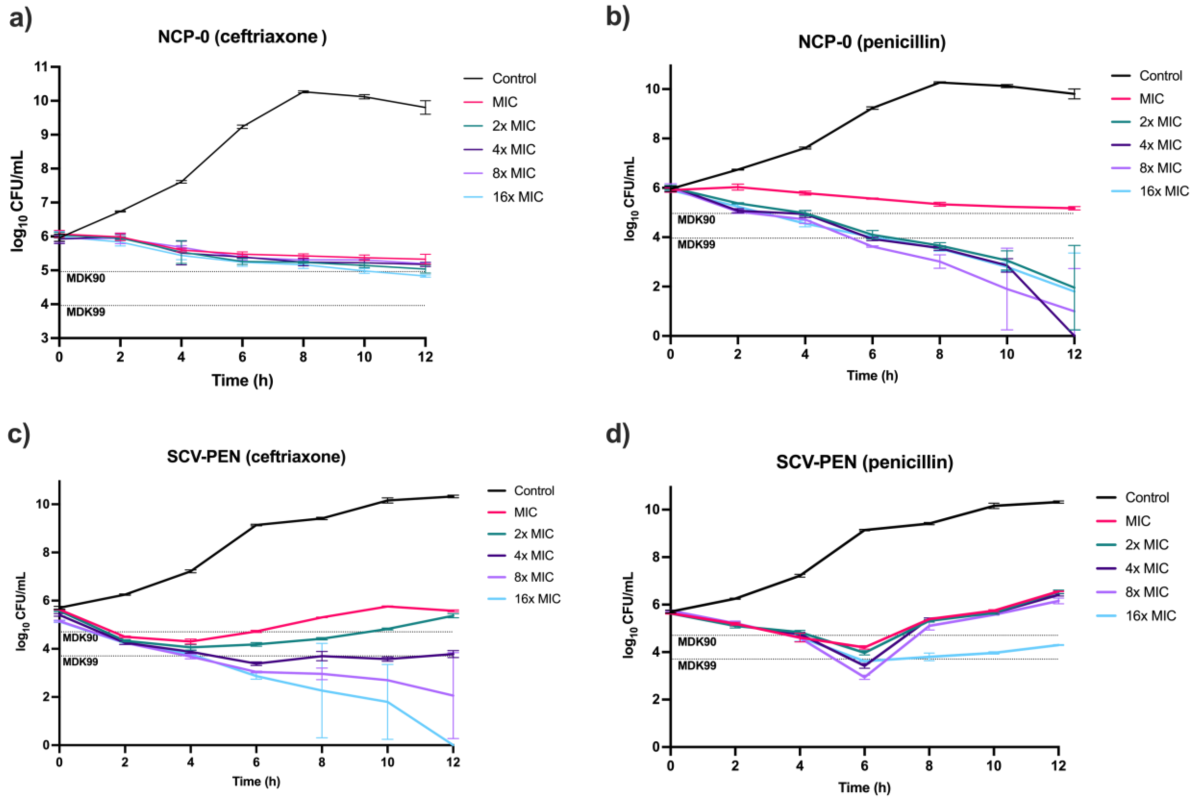

Figure S4 Time–kill kinetics of *E. faecalis* normal colony phenotype NCP-0 under ceftriaxone (a) and benzylpenicillin (b) exposure and small colony variants SCV-PEN under ceftriaxone (c) and benzylpenicillin (d) exposure. Time–kill curves were generated to determine MDK_{90} and MDK_{99} values at increasing antibiotic concentrations (MIC, 2× MIC, 4× MIC, 8× MIC, and 16× MIC) over a 12 h period. Viable counts are expressed as log_{10} CFU/mL (mean ± SD) from independent biological replicates.

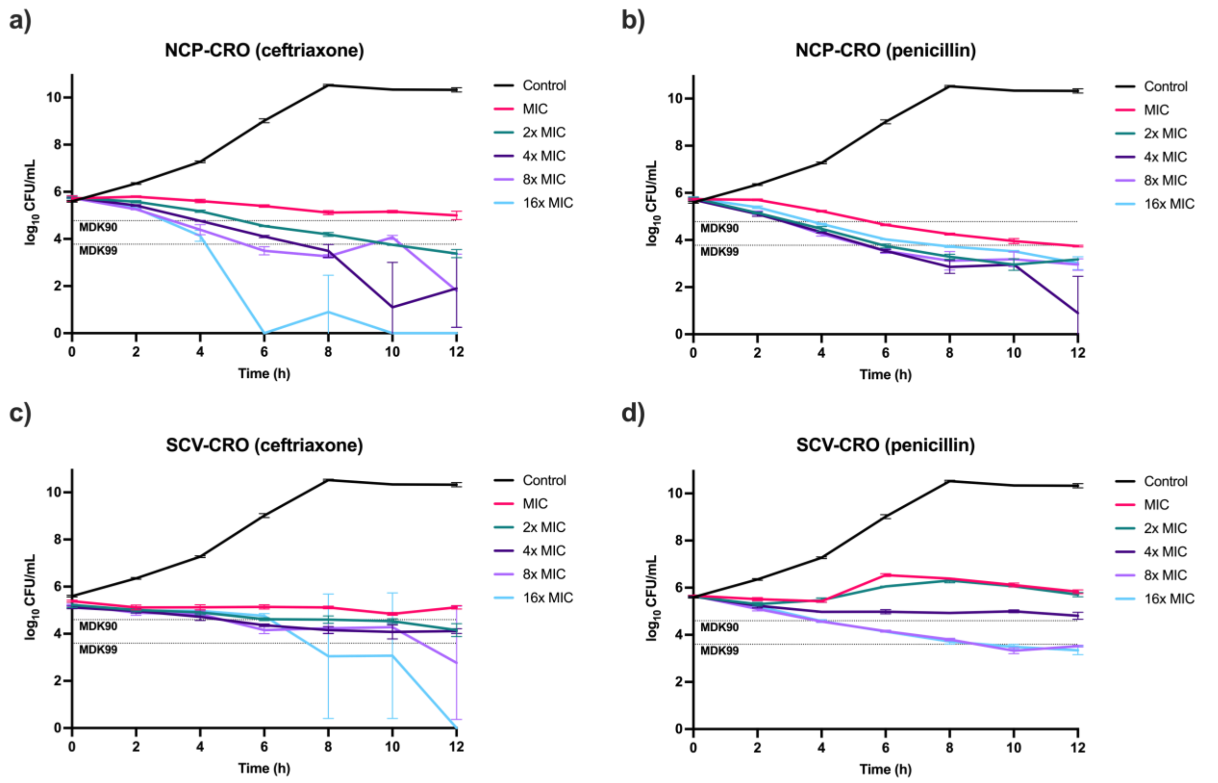

Figure S5 Time–kill kinetics of *E. faecalis* normal colony phenotype NCP-CRO under ceftriaxone (a) and benzylpenicillin (b) exposure and small colony variants SCV-CRO under ceftriaxone (c) and benzylpenicillin (d) exposure. Time–kill curves were generated to determine MDK$_{90}$ and MDK$_{99}$ values at increasing antibiotic concentrations (MIC, 2× MIC, 4× MIC, 8× MIC, and 16× MIC) over a 12 h period. Viable counts are expressed as log$_{10}$ CFU/mL (mean ± SD) from independent biological triplicates.

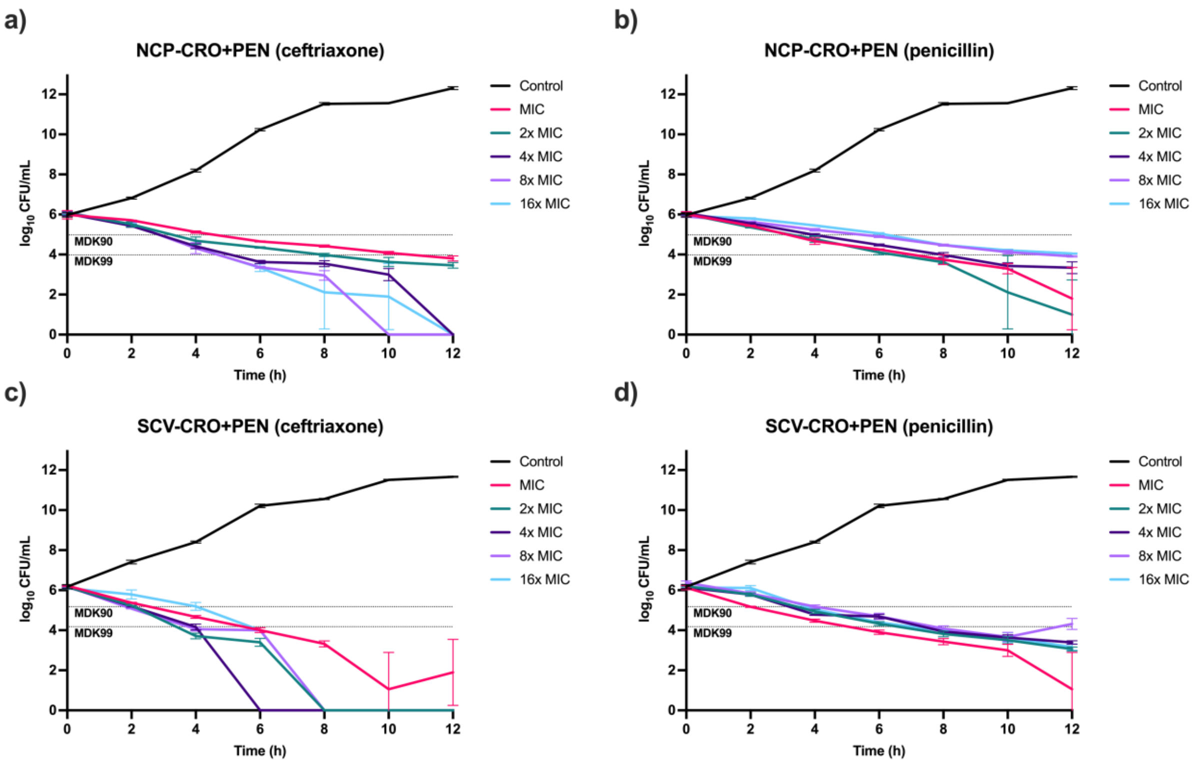

Figure S6 Time–kill kinetics of *E. faecalis* normal colony phenotype NCP-CRO+PEN under ceftriaxone (a) and benzylpenicillin (b) exposure and small colony variants SCV-CRO+PEN under ceftriaxone (c) and benzylpenicillin (d) exposure. Time–kill curves were generated to determine MDK_{90} and MDK_{99} values at increasing antibiotic concentrations (MIC, 2× MIC, 4× MIC, 8× MIC, and 16× MIC) over a 12 h period. Viable counts are expressed as log_{10} CFU/mL (mean ± SD) from independent biological replicates.

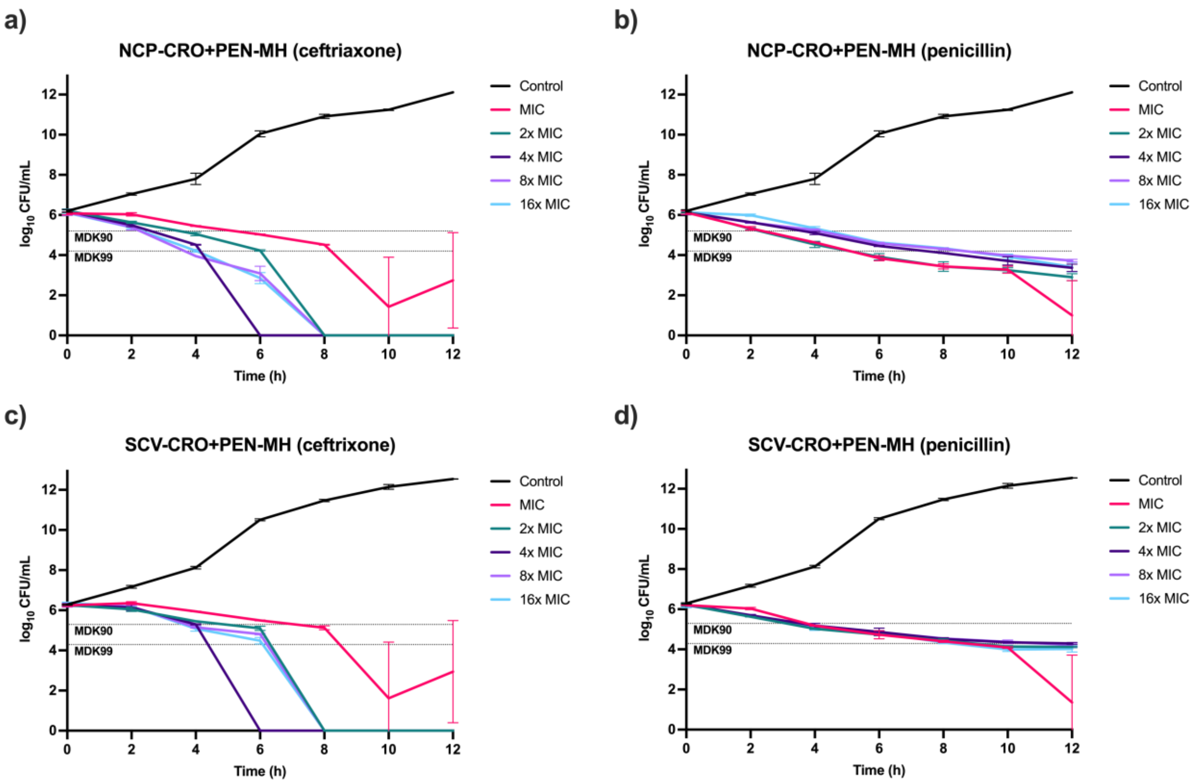

Figure S7 Time–kill kinetics of *E. faecalis* normal colony phenotype NCP-CRO+PEN-MH under ceftriaxone (a) and benzylpenicillin (b) exposure and small colony variants SCV-CRO+PEN-MH under ceftriaxone (c) and benzylpenicillin (d) exposure. Time–kill curves were generated to determine MDK_{90} and MDK_{99} values at increasing antibiotic concentrations (MIC, 2× MIC, 4× MIC, 8× MIC, and 16× MIC) over a 12 h period. Viable counts are expressed as $\log_{10}$ CFU/mL (mean ± SD) from independent biological replicates.

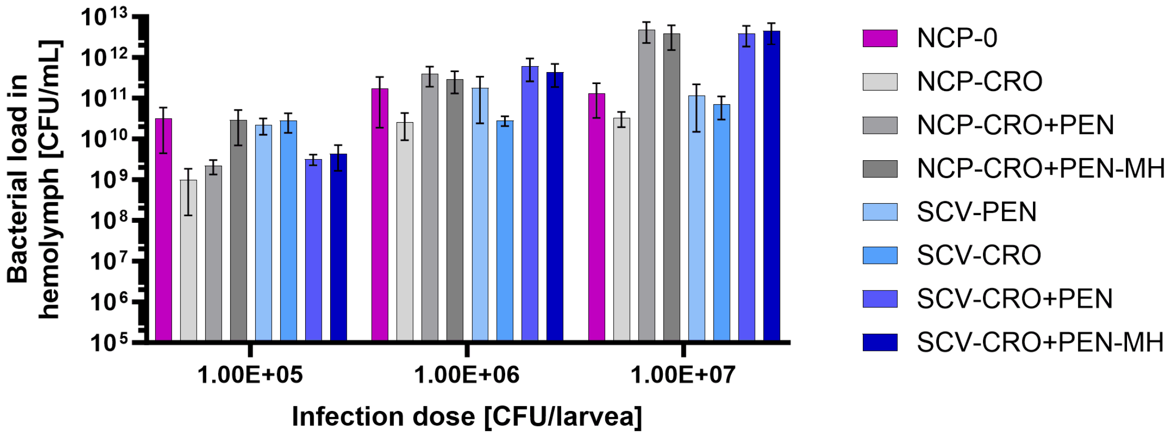

Figure S8 Endpoint bacterial burden in *G. mellonella* larvae 72 h post infection. Larvae were infected with the indicated *E. faecalis* variants and bacterial burden was determined at death time point or latest after 72 h post infection by quantifying colony-forming units (CFU) from hemolymph samples (N = 10 larvae per variant). The measured values represent the total bacterial population recovered, including both SCV and NCP phenotypes. The data is presented as measn and standard deviations.

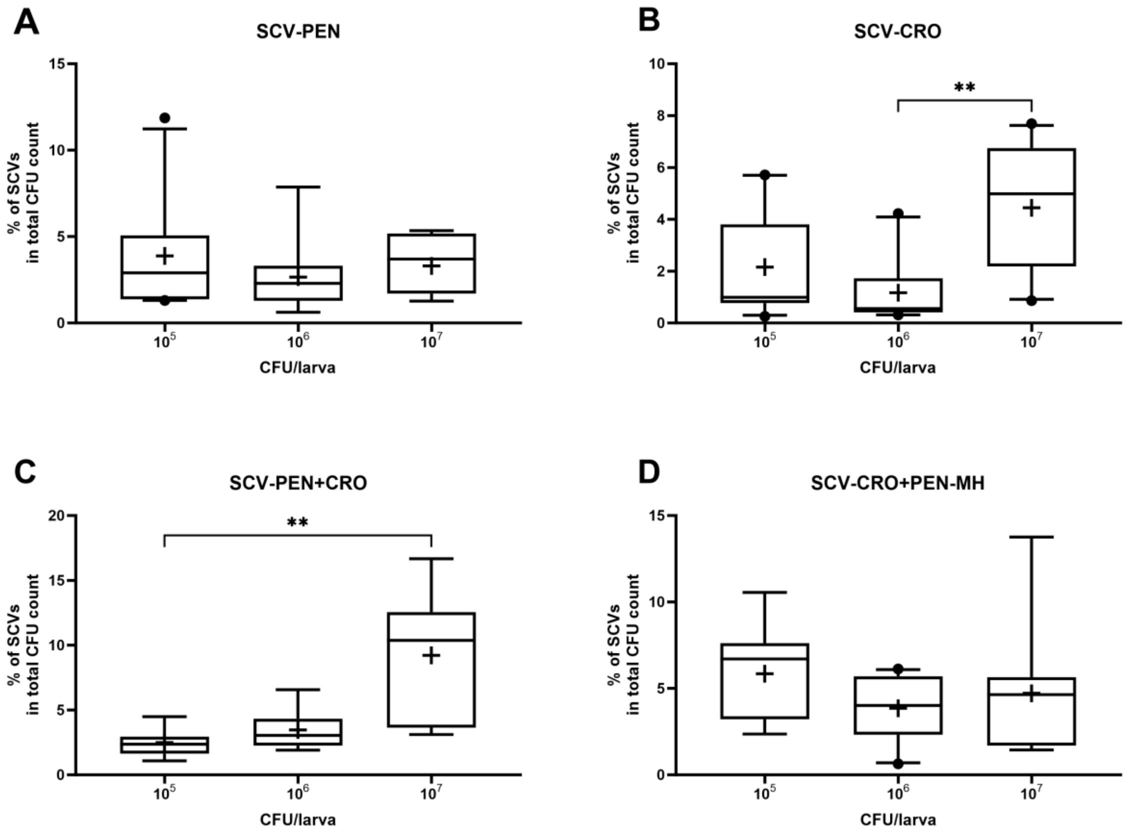

Figure S9 Proportion of SCV among the total CFU recovered from larval hemolymph after infection with the respective SCV phenotype. Hemolymph was collected either at 72 h post infection from surviving larvae or immediately after scoring from larvae that died earlier (N=10 per variant).
